# Supplementary material for: Splice-Junction-Based Mapping of Alternative Isoforms in the Human Proteome
Source: Cell Rep. Author manuscript; Available in PMC 2020 Jan 15. (PMC6961840; doi:10.1016/j.celrep.2019.11.026)
Supplement: 2 [file NIHMS1546469-supplement-2.zip › DF1/PXD010154/Liver/Liver_18_MAP4_MYHDDDLADLVFPSSATADTSIFAGQNDPLKDSYVPLELAK.pdf]

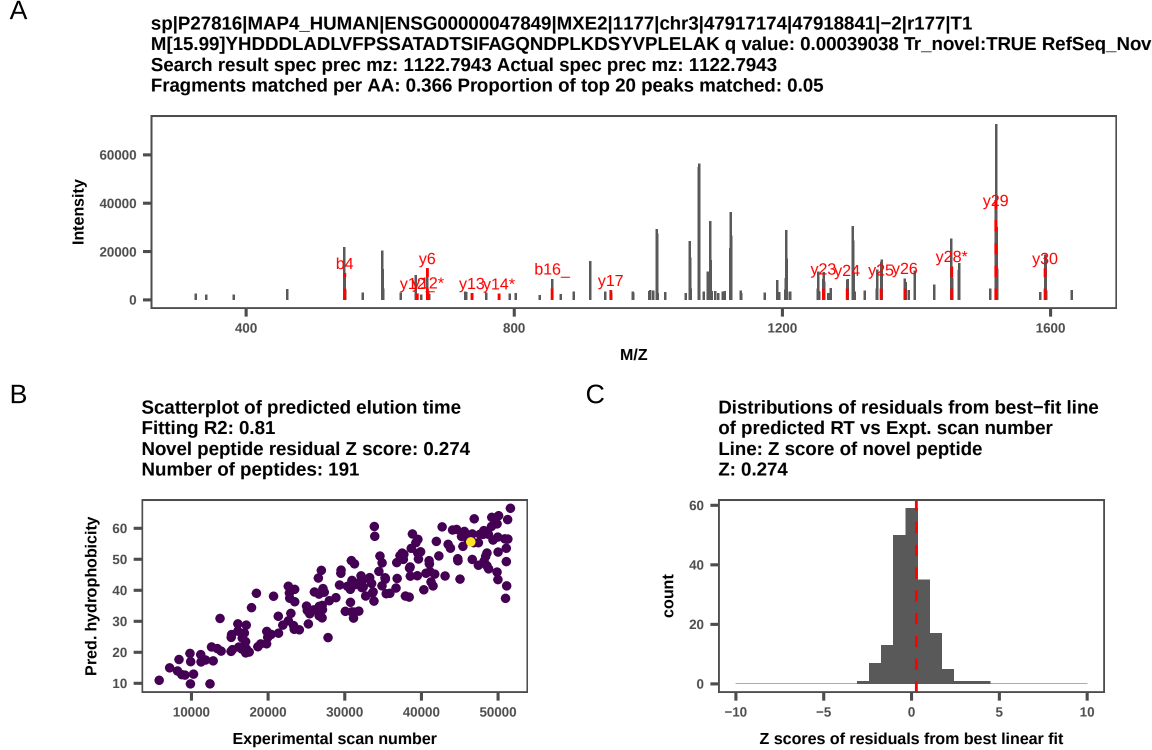
A
sp|P27816|MAP4_HUMAN|ENSG00000047849|MXE2|1177|chr3|47917174|47918841|−2|r177|T1
M[15.99]YHDDDLADLVFPSSATADTSIFAGQNDPLKDSYVPLELAK q value: 0.00039038 Tr_novel:TRUE RefSeq_Nov
Search result spec prec mz: 1122.7943 Actual spec prec mz: 1122.7943
Fragments matched per AA: 0.366 Proportion of top 20 peaks matched: 0.05
Intensity
60000
40000
20000
0
b4
y6
y11
y12*
y13
y14*
b16_
y17
y23
y24
y25
y26
y28*
y29
y30
400
800
1200
1600
M/Z
B
Scatterplot of predicted elution time
Fitting R2: 0.81
Novel peptide residual Z score: 0.274
Number of peptides: 191
Pred. hydrophobicity
60
50
40
30
20
10
10000
20000
30000
40000
50000
Experimental scan number
C
Distributions of residuals from best−fit line
of predicted RT vs Expt. scan number
Line: Z score of novel peptide
Z: 0.274
count
60
40
20
0
−10
−5
0
5
10
Z scores of residuals from best linear fit
